# Prediction models for amputation after diabetic foot: systematic review and critical appraisal

**DOI:** 10.1186/s13098-024-01360-6

**Published:** 2024-06-10

**Authors:** Jingying Huang, Jin Yang, Haiou Qi, Miaomiao Xu, Xin Xu, Yiting Zhu

**Affiliations:** 1 Postanesthesia Care Unit, Sir Run Run Shaw Hospital, Zhejiang University School of Medicine, Hangzhou, China; 2 Nursing Department, Sir Run Run Shaw Hospital, Zhejiang University School of Medicine, Hangzhou, China; 3 Orthopedics Department, Sir Run Run Shaw Hospital, Zhejiang University School of Medicine, Hangzhou, China; 4 Operating Room, Sir Run Run Shaw Hospital, Zhejiang University School of Medicine, Hangzhou, China

**Keywords:** Diabetic foot, Amputation, Prediction model, Systematic review

## Abstract

**Background:**

Numerous studies have developed or validated prediction models aimed at estimating the likelihood of amputation in diabetic foot (DF) patients. However, the quality and applicability of these models in clinical practice and future research remain uncertain. This study conducts a systematic review and assessment of the risk of bias and applicability of amputation prediction models among individuals with DF.

**Methods:**

A comprehensive search was conducted across multiple databases, including PubMed, Web of Science, EBSCO CINAHL Plus, Embase, Cochrane Library, China National Knowledge Infrastructure (CNKI), Wanfang, Chinese Biomedical Literature Database (CBM), and Weipu (VIP) from their inception to December 24, 2023. Two investigators independently screened the literature and extracted data using the checklist for critical appraisal and data extraction for systematic reviews of prediction modeling studies. The Prediction Model Risk of Bias Assessment Tool (PROBAST) checklist was employed to evaluate both the risk of bias and applicability.

**Results:**

A total of 20 studies were included in this analysis, comprising 17 development studies and three validation studies, encompassing 20 prediction models and 11 classification systems. The incidence of amputation in patients with DF ranged from 5.9 to 58.5%. Machine learning-based methods were employed in more than half of the studies. The reported area under the curve (AUC) varied from 0.560 to 0.939. Independent predictors consistently identified by multivariate models included age, gender, HbA1c, hemoglobin, white blood cell count, low-density lipoprotein cholesterol, diabetes duration, and Wagner’s Classification. All studies were found to exhibit a high risk of bias, primarily attributed to inadequate handling of outcome events and missing data, lack of model performance assessment, and overfitting.

**Conclusions:**

The assessment using PROBAST revealed a notable risk of bias in the existing prediction models for amputation in patients with DF. It is imperative for future studies to concentrate on enhancing the robustness of current prediction models or constructing new models with stringent methodologies.

**Supplementary Information:**

The online version contains supplementary material available at 10.1186/s13098-024-01360-6.

## Introduction

In recent years, with lifestyle improvements and increased life expectancy, the prevalence of diabetes mellitus (DM) has surged [1]. Diabetic foot (DF), being one of the most prevalent, severe, and costly complications of DM, is primarily characterized by skin infections, ulcers, or destruction of deep foot tissues below the ankle joint. It is commonly associated with neuropathy or vascular disorders in the lower extremities, and in severe cases, it may involve muscles and bones [2]. It is estimated that approximately 537 million people worldwide have diabetes, and 19% to 34% of them will experience diabetic foot ulcers (DFU) to varying degrees during their lifetime [3]. Around 20% of DFU patients may require lower limb amputations, which can be either minor (below the ankle joint) or major (above the ankle joint), and sometimes both [4]. Previous research indicated that every 30 s, one lower limb was amputated due to diabetes, with an average annual cost of $8659 per patient for DF care [5]. The 5-year mortality rates for DFU, minor amputations, and major amputations were reported to be 30.5%, 46.2%, and 56.6%, respectively [6]. In addition to DFU’s considerable impact on mortality, DFU is also associated with devastating financial, emotional, and psychological burden [7].

Recent research suggests that around 75% of DF patients ultimately undergo amputation, primarily due to lower limb vascular disease, nerve abnormalities, poor blood sugar control, and concurrent ulcer complications [8]. Various factors, including age, gender, ulcer depth, infection severity, local ischemia, osteomyelitis, diabetes duration, neuropathy, and blood sugar control, are considered potential predictors for DFU amputation. However, there remains a lack of complete understanding regarding the most significant factors and their respective impact on the risk of amputation [9]. A significant factor contributing to DF issues is the improper wearing of shoes and socks [10]. The management of the unaffected foot emerges as a pivotal aspect of self-care for those who have undergone amputation due to DF. Dealing with DF is a prolonged and recurrent treatment process, currently lacking an effective cure. Key to successful DFU management lies in regular screening, identifying all risk factors for DF, and making corrections whenever possible [11]. The International Working Group on the Diabetic Foot (IWGDF) emphasizes that prevention, early diagnosis, active screening, and self-management of DFU can potentially avert over 90% of amputations [10]. Beyond symptomatic treatment, there is a critical need to empower patients with enhanced self-management skills. This encompasses taking subjective initiative in disease management, health guidance, care coordination, physical care, blood sugar monitoring, psychological adjustment, nutritional intervention, and exercise compliance. Ultimately, this comprehensive approach aims to significantly reduce the occurrence of amputations [12].

Given the high prevalence of DFU, their substantial socioeconomic burden, and the profound impact on patient autonomy and quality of life, it is imperative to identify early predictors of DF amputation and promptly recognize populations at risk that may benefit from early prevention and targeted interventions. Furthermore, efforts should be made to avoid unnecessary amputations due to variations in clinical expertise and insufficient judgment, preserving the integrity of the limbs and preventing catastrophic consequences [13]. The early identification of potential amputations also allows for an extended period to implement pre-operative rehabilitation programs, thereby further enhancing patient outcomes [14]. Evidence indicates that early identification may improve patient acceptance of prosthetic limb usage and reduce complications on the same side of the leg [15]. Moreover, early prediction of amputation can assist multidisciplinary teams in offering emotional and psychological support to patients before undergoing surgery. This process fosters patient awareness and engagement in treatment decisions, ultimately improving disability acceptance in diabetic amputees [16]. Many existing DFU classification systems [17, 18], such as Wagner’s classification [19], University of Texas classification (TUC), WIFi (Wound, Ischaemia, Foot infection) [20] or site, ischaemia, neuropathy, bacterial infection, and depth classification, are commonly utilized tools for selecting treatment options and predicting the risk of amputation in DFU patients. Although these DFU classification systems have the ability to predict amputation, a widely accepted gold standard has yet to be established. These systems mostly based on clinical subjectivity experience or expert consensus, lacking robust support from objective data or validation from effective external data. Accurate prediction of amputation in complex clinical Settings remains a challenging issue [21]. Additionally, these systems do not fully assess the impact of demographics, clinical or laboratory data, medical history, foot condition, and other risk factors on amputation rates, making them less sensitive and specific.

Risk prediction models amalgamate various factors to assess the likelihood of specific conditions (diagnostic model) or events occurring in the future (prognostic model). This is primarily achieved through the utilization of regression equations, nomographs, or innovative approaches grounded in artificial intelligence [22–24]. In recent years, an increasing number of studies have focused on developing or validating predictive models to estimate the risk of DF amputation, however, the quality and applicability of model development has not been systematically evaluated, leaving healthcare professionals uncertain about which model to recommend and for whom or under what circumstances. Therefore, this study aims to systematically analyze and evaluate predictive models for the occurrence of amputation in DF cases, with the aim of providing valuable insights to inform future studies in this field.

## Materials and methods

### Study design

This study was registered in PROSPERO (Registration ID: CRD42023493907) prior to commencing the search.

### Search strategy

We performed a thorough computerized search of multiple databases, including PubMed, Web of Science, EBSCO CINAHL Plus, Embase, Cochrane Library, China National Knowledge Infrastructure (CNKI), Wanfang, Chinese Biomedical Literature Database (CBM), and Weipu (VIP). The search encompassed the period from the inception of the databases to December 24, 2023. The search strategy employed in this study involved a comprehensive approach. Medical subject headings (MeSH) and free words were combined in the titles, abstracts, and keywords to ensure a thorough search. Additionally, a retrieval filter based on the prediction model was used, along with manual retrieval and citation retrieval methods. The following keywords were used to conduct a basic search: “diabetes mellitus,” “foot ulcer,” “amputation,” “Prognostic,” “rule,” “Predict*,” “Validat*,” “risk assessment,” “risk score,” and “algorithm”. (Detailed information regarding the search strategies can be found in the Supplementary materials). Further studies were identified by reviewing the reference lists of the retrieved studies and review articles.

### Study selection

Two researchers, HJY and YJ, independently conducted the study selection based on titles and abstracts, followed by a thorough evaluation of the full texts. All prediction modeling studies, whether with or without external validation, and external validation studies, whether with or without model updating, were included if they satisfied the predefined inclusion criteria outlined in PICOTS.

P (Population): the population of interest comprises patients diagnosed with DF, regardless of whether they have type 1 or type 2 diabetes (T2D), and who were aged above 18 years old.

I (Intervention model): studies focus on prediction models that were internally or externally validated, specifically for prognostic models predicting the risk of amputation after diabetic foot.

C (Comparator): not applicable.

O (Outcome): outcome was defined as amputation, including major or minor amputations, following a diagnosis of DF or DFU.

T (Timing): outcomes were predicted after the diagnosis of DF or DFU, with no restriction on the time frame of the prediction.

S (Setting): the intended use of the prediction model was to perform risk stratification in the assessment of amputation development in medical institutions, such that preventive measures could be used.

We incorporated all original and peer-reviewed development and validation studies, encompassing both English and Chinese publications. Acceptable study types included cohort studies, cross-sectional studies, randomized controlled trials, and case–control studies, with eligibility for studies published in either Chinese or English. Exclusion criteria involved studies related to animals, non-human samples, and non-first amputation scenarios. Additionally, informally published literature, limited methodological data availability, studies conducted at the cellular and molecular levels, prediction models based on virtual data, models with fewer than two predictive variables, repeated publications, poor-quality literature, and studies lacking available original data were excluded. The inability to access the full text of the literature also served as an exclusion criterion.

### Data extraction

Two investigators independently conducted the extraction of data, and a third investigator crosschecked the results. Any disparities or differences were resolved through discussions among the researchers or by consulting with professionals. The Critical Appraisal and Data Extraction for Systematic Reviews of Prediction Modelling Studies (CHARMS) checklist was used to guide the data extraction [25]. The collected data encompass a range of elements, including key characteristics of all included literature (such as publication year, study country, study design, population, data source, and follow-up time), details about predicted outcomes (including diagnostic criteria), information about the establishment of prediction models (such as the number of candidate variables, the processing method for continuous variables, sample size, number of result events, number of missing data, processing method, model establishment details, and variable selection method), as well as insights into model performance and prediction factors (covering model performance evaluation, validation methods, main prediction factors, model presentation, applicability, and limitations).

### Assessment of methodological quality

Two reviewers (HJY and YJ) independently conducted a critical appraisal of each article to assess the risk of bias and the applicability of the models to the intended population and setting. In the event of any disagreements, a third reviewer (XMM) provided input. This assessment was performed using an Excel file based on the Prediction Study Risk of Bias Assessment Tool (PROBAST) [26]. This tool comprises 20 signaling questions distributed among four primary domains, including participants, predictors, outcome, and analysis. Each signaling question can be answered yes (Y), probably yes (PY), no (N), probably no (PN), or no information (NI). In a domain, all answers must be Y or PY to be considered “low risk.” If at least one signaling question is answered N or PN, it is categorized as “high risk.” When a signaling question is judged as NI and all other signals indicate “low risk,” the domain is classified as “unclear.” Applicability is assessed as either “good applicability,” “poor applicability,” or “unclear applicability.” When all domains are evaluated as low risk of bias or good applicability, the overall judgment is low risk of bias or good applicability. If anyone domain is rated as high risk of bias or poor applicability, the overall assessment becomes high risk of bias or poor applicability. In cases where bias risk in a domain or applicability is unclear, but the bias risk in other domains is low or applicability is good, the overall bias risk is considered unclear, or the applicability is deemed unclear.

### Analysis

The model’s discrimination was assessed using the area under the ROC curve (AUC) [27]. Software GraphPad Prism 9.0 was utilized for analyzing the AUC values of the model. We classified AUC values within the range of 0.5–0.7 as indicating poor discrimination, 0.7–0.8 as moderate discrimination, 0.8–0.9 as representing good discrimination, and 0.9–1.0 as indicating excellent discrimination. Additionally, predictors distribution and a percentage stacked chart pertaining to the risk of bias and applicability assessment were created using office software Excel.

## Results

### Study selection

**Fig. 1:**
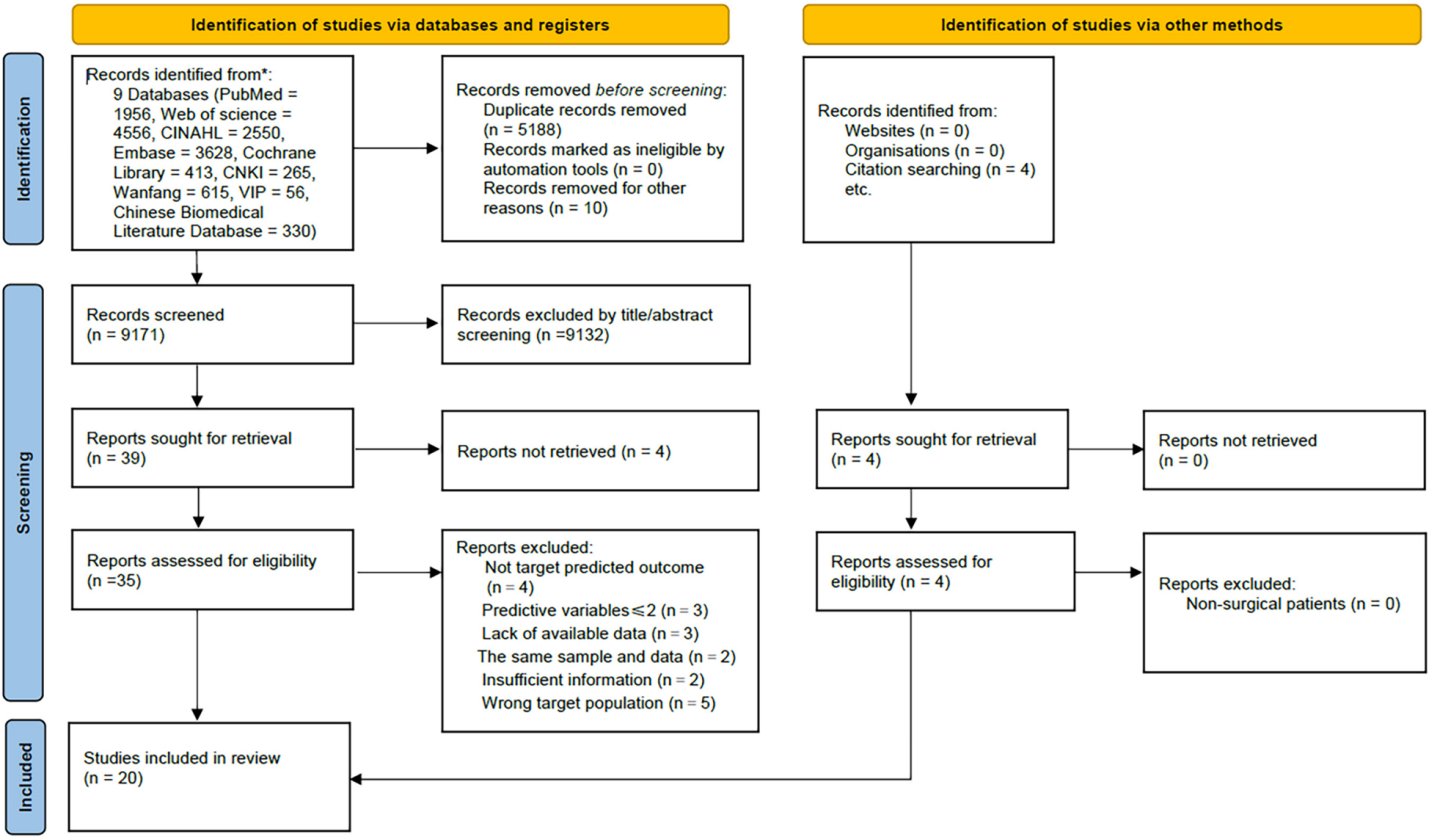
PRISMA 2020 flow diagram for systematic review

Initially, 14,369 records were retrieved through the system. After removing duplicated studies, 9181 articles remained. Upon reviewing titles and abstracts, 9132 articles unrelated to the research topic were excluded. Furthermore, we identified four studies through citation searches of relevant systematic reviews and conducted full-text readings for 39 articles. Among these, we excluded five studies targeting populations with diabetes or peripheral artery disease (PAD), five studies predicting outcomes such as re-amputation or considering indicators like death as adverse events, three studies with fewer than two predictors, two studies with duplicate sample data, and five studies with missing important data or lacking model performance evaluations. Ultimately, 20 articles were included in this review. Figure 1 illustrated a flowchart depicting the Preferred Reporting Items for Systematic Reviews and Meta-Analyses (PRISMA) 2020 guidelines, outlining the comprehensive search process and its outcomes.

### Characteristics of the included studies

Among the included literatures, 17 [17, 18, 22, 23, 28–40] were in English and three [24, 41, 42] were in Chinese, with 12 publications [22–24, 28–35, 41] within the last 5 years. Covering studies conducted in nine countries: 10 [22–24, 28, 29, 31, 32, 34, 41, 42] from China, two each from the United States [30, 39] and India [36, 37], and one each from Germany [35], the Philippines [33], South Korea [17], Portugal [17], the Netherlands [38], and Spain [40]. Seven studies [18, 33–36, 38, 42] were prospective, while the remainder were retrospective. Three papers [31, 40, 41] focused on predictive models for DF amputation in T2D patients. Five studies [28–30, 38, 39] involved multiple centers, while the remaining were conducted at single centers. Two studies [30, 33] concentrated on major amputation as the primary outcome, one [29] on minor amputation, and four studies [ 18, 23, 35, 38] simultaneously predicted two different outcome indicators (e.g., amputation and minor amputation, amputation and major amputation, minor amputation and major amputation). The remaining studies focused on any form of amputation, with one study [37] reporting simple and complex amputation prediction models. Sample sizes ranged from 23 to 32,685 participants across the studies, with follow-up periods ranging from 3 months to 3.5 years. An overview of the essential study characteristics can be found in Table 1.

**Table 1:** Basic characteristics of the included studies

Author (year), country	Study design	Participants	Data source	Outcomes to be predicted	Amputation cases/sample size (%)	Follow-up time
Chen et al. [22] (2023), China	Retrospective cohort	DF, unspecified diabetes	EMR of one hospital (2015–2020) + telephone	Amputation/death related to DF	25/200 (12.5%)	3 years
Li et al. [28] (2023), China	Retrospective cohort	Inpatients with DF	Healthcare Big Data Platform of two hospitals (2014–2021)	Amputation/all-cause death	15/175 (8.6%)	2 years
Yang [41] (2023), China	Retrospective study	T2D with DFU	EMR of one hospital (2015–2020)	Amputation	213/364 (58.5%)	–
Stefanopoulos et al. [30] (2022), America	Retrospective study	Adult inpatients with DFU	Nation inpatient sample of HCUP (2008–2014)	Major lower extremity amputation	1 928/32 685 (5.9%)	–
Wang et al. [29] (2022), China	Retrospective study	Texas grade 3 (UTC 3) DFU patients	EMR of two hospitals (2018–2019)	Minor amputation	75/362^a^ (20.7%)	–
Xie et al. [23] (2022), China	Retrospective cohort	Adult inpatients with DFU	HER of one hospital (2009–2020)	Minor amputation/major amputation	Minor: 71/618 (11.5%); major: 47/618 (7.6%)	–
Du et al. [32] (2021), China	Retrospective case control	DFU diagnosed and conformed to WIFI wound classification, grade 1–3	HER of one hospital, COVID-19 pandemic, post-lockdown (2020)	In-hospital amputation/death	6/23 (26.1%)	NA
Li [24] (2021), China	Retrospective case control	DF, unspecified diabetes	HER of one hospital	Amputation	118/618 (19.1%)	NA
Peng et al. [31] (2021), China	Retrospective case control	Adults with T2D were diagnosed DFU	EMR of two departments in one hospital (2015–2019)	Amputation	58/125 (46.4%)	NA
Hüsers et al. [58] (2020), Germany	Prospective cohort	Inpatients or outpatients DM with DFU	EMR of one hospital (2013–2019)	Any-amputation^b^/major amputation	Any: 75/237 (31.6%) Major: 29/237 (12.2%)	6 months
Lin et al. [34] (2020), China	Prospective cohort	Inpatients with DF	EMR of one hospital (2018) + telephone	Amputation/death related to DF	–/200 (–)	3 years
Vera-Cruz et al. [33] (2020), Philippines	Prospective cohort	19 years or older and diagnosed with DF	Interview + physical examination + EMR	Major amputation	17/63 (27.0%)	1 year
Chetpet et al. [36] (2018), India	Prospective cohort	Adult inpatients or outpatient with DFU	One hospital (2015–2016)	Amputation	44/150 (29.3%)	1 year
Chen [42] (2018), China	Prospective cohort	Inpatients with DF	EMR of one hospital (2014–2016) + telephone	Amputation/death related to DF	37/273 (13.6%)	3 years
Jeon et al. [17] (2017), Korea	Retrospective cohort	Inpatients with DFU (at least to Wagner stage 1)	Chart + photographic + EMR (2010–2014)	Amputation	67/137 (48.9%)	–
Kasbekar et al. [37] (2017), India	Retrospective cohort	Inpatients with DF	EMR of one hospital (2011–2013)	Amputation	83/301 (27.6%)	–
Monteiro-Soares [18] (2015), Portugal	Prospective cohort	Outpatients with DM and active foot ulcer	One DF outpatient clinic (2010–2013)	Amputation/major amputation	68/293 (23.2%) 19/29 3(6.5%)	≥ 3 months
Pickwell et al. [38] (2015), Netherlands	Prospective cohort	DM presenting with a new foot ulcer	14 DF clinics in 10 European countries (2003–2004)	Amputation/minor amputation^c^	159/575 (27.7%) 103/575 (17.9%)	1 year
Lipsky et al. [13] (2011), America	Retrospective case control	Inpatients with DF infection	A database of 97 acute-care hospitals (2003–2007)	In-hospital amputation	647/3018 (21.4%)	NA
Barberan et al. [40] (2010), Spain	Retrospective cohort	Inpatient T2D with acute DF infection	Clinical records of one hospital	Amputation	26/78 (33.3)	≥ 3.5 year

Minor amputation was defined as any amputation below the ankle, whereas major amputation was defined as amputation above the ankle. Amputation was defined as a minor or major amputation

*DF* diabetic foot, *EMR* electronical medical records, *T2D* type 2 diabetes, *DFU* diabetic foot ulcers, *NA* not applicable, *DM* diabetes mellitus, *HCUP* healthcare cost and utilization project, *HER* electronic health records, *UTC* University of Texas classification

^a^Oversampling was used to expand the sample to 573 cases

^b^Excluding lesser toes; “–” indicated not report

^c^Amputations proximal to and including the hallux

### Basic features of prediction model

A total of 54,265 DF patients were included in these studies. Amputation occurred in 3779 patients, with a prevalence of 5.9–58.5%. Three of the included papers utilized external data to validate the predictive performance of existing amputation scoring systems [17, 18, 33], while the remaining studies focused on developing new models. The range of candidate factors considered in each study varied from seven to 44, with the events per variable (EPV) spanning from 0.194 to 53.556. Among the 17 studies dedicated to model development, five opted for traditional logistic regression (LR) [31, 36, 38–40], four employed a single machine learning (ML) method [23, 28, 35, 37], and eight utilized multiple ML techniques [22, 24, 29, 30, 32, 34, 41, 42]. The most prevalent ML method was random forest (RF), used in five studies, followed by extreme gradient boosting (XGBoost) and support vector machine (SVM), each appearing in four studies. Across the 20 papers, a total of 77 prediction models were constructed, with 20 optimal models highlighted across 17 model development studies. Notably, in all five studies, data with incomplete clinical information were excluded, yet details regarding the number of missing values and the handling methods were not explicitly mentioned [22, 28–30, 37]. Regarding model evaluation, only 11 studies appropriately assessed differentiation and calibration. Thirteen model development studies employed internal validation techniques, with seven using Bootstrap or K-fold cross-validation methods [23, 24, 29, 32, 41], while four lacked a formal model validation process [34, 35, 37, 39]. In terms of model presentation, Stefanopoulos et al. [30] and Wang et al. [29] developed web-based risk calculators for clinical application and dissemination, Li [24] created amputation risk assessment and prediction software, and Peng et al. [31] transformed the complex regression equation into a visual line graph model. Xie et al. [31] utilized SHapley Additive Explanations (SHAP) to visually illustrate the contribution of each feature to the model’s predictions. Three models were based on equations [22, 35, 41] and scoring systems [36, 39, 40], whereas in other studies, the model presentation formats were not specified. Table 2 displayed the overview of model constructed for the included prediction models.

**Table 2:** Key characteristics for constructing amputation prediction models in diabetic foot patients

First author year	Candidate variables			Missing data		Variable selection methods None	Type of validation	Model evaluation	Calibration method
	No	Continuous variables processing method	EPV	No	Processing methods				
Chen 2023	14	Remain unaltered	1.786	–	Missing were excluded, analysis with complete data	CR	Internal	Random split validation	None
Li 2023	31	Remain unaltered	0.484	–	Missing were excluded, analysis with complete data	VIMP	Internal	Bootstrap	H–L test
Yang 2023	32	Remain unaltered	6.656	< 10% of quantitative data	Missing values > 40% were deleted, Median was used for missing quantitative data	FSR, Pruning algorithm	Internal	Tenfold cross validation	H–L test
Stefanopoulos 2022	36	Some converted to categorical variables	53.556	–	Missing were excluded, analysis with complete data	Lasso regression	Internal	Random split validation	None
Wang 2022	21	All converted to categorical variables	3.571	–	Missing were excluded, analysis with complete data	LR	Internal	Tenfold cross validation	None
Xie 2022	37	Remain unaltered	1.919 0.270	–	Model automatically handle	None	Internal	Fivefold cross validation	H–L test, calibration curve
Du 2021	31	Some converted to categorical variables	0.194	–	–	None	Internal	Threefold cross validation	None
Li 2021	44	Z-score standardization	2.682	70	Delete of features with > 60% missing values. The rest were imputed using median, mean, mode, fixed value, or KNN	RF-RFE, mRMR, JMI, JMIM, original	Internal	Fivefold cross validation	None
Peng 2021	21	Remain unaltered	2.762	–	Sever missing values were excluded	FSR	Internal	Bootstrap	H–L test, Calibration curve
Hüsers 2020	7	Remain unaltered	10.714 4.143	LTFU:16	Analysis with complete data	–	None	None	None
Lin 2020	33	Remain unaltered	–	–	Analysis with complete data	CR	Internal	Random split validation	None
Vera-Cruz 2020	NA	Some converted to categorical variables	NA	None	–	NA	External	Spatial validation	None
Chetpet 2018	13	All converted to categorical variables	3.384	LTFU:21	Analysis with complete data	–	None	None	None
Chen 2018	33	Some converted to categorical variables	1.121	–	–	CR	Internal	Random split validation	None
Jeon 2017	NA	Remain unaltered	NA	LTFU:21	Analysis with complete data	NA	External	Spatial validation	None
Kasbekar 2017	17	Remain unaltered	4.882	–	Missing were excluded, analysis with complete data	–	Internal	Random split validation	None
Monteiro-Soares 2015	NA	Remain unaltered	NA	LTFU: 9 Miss: 223	Missing were excluded, analysis with complete data	NA	External	Spatial validation	None
Pickwell 2015	20	Some converted to categorical variables	7.950 5.150	–	–	FSR	None	None	None
Lipsky 2011	33	Some converted to categorical variables	19.606	–	–	SR	Internal	Random split validation	H–L test
Barberan 2010	19	Remain unaltered	1.368	–	–	UA	None	None	None

“–” Indicated not reported

*EPV* events per variable, *CR* Cox regression, *VIMP* variable importance, *H–L test* Hosmer–Lemeshow goodness of fit test, *FSR* forward stepwise regression, *LR* logistic regression, *KNN* K-Nearest Neighbor, *RF-RFE* random forest recursive feature elimination, *LTFU* lost to follow-up, *mRMR* minimum redundancy maximum relevance, *JMI* joint mutual information, *JMIM* joint mutual information maximization, *SR* stepwise regression, *UA* univariate analysis

### Model performance and predictors

**Fig. 2:**
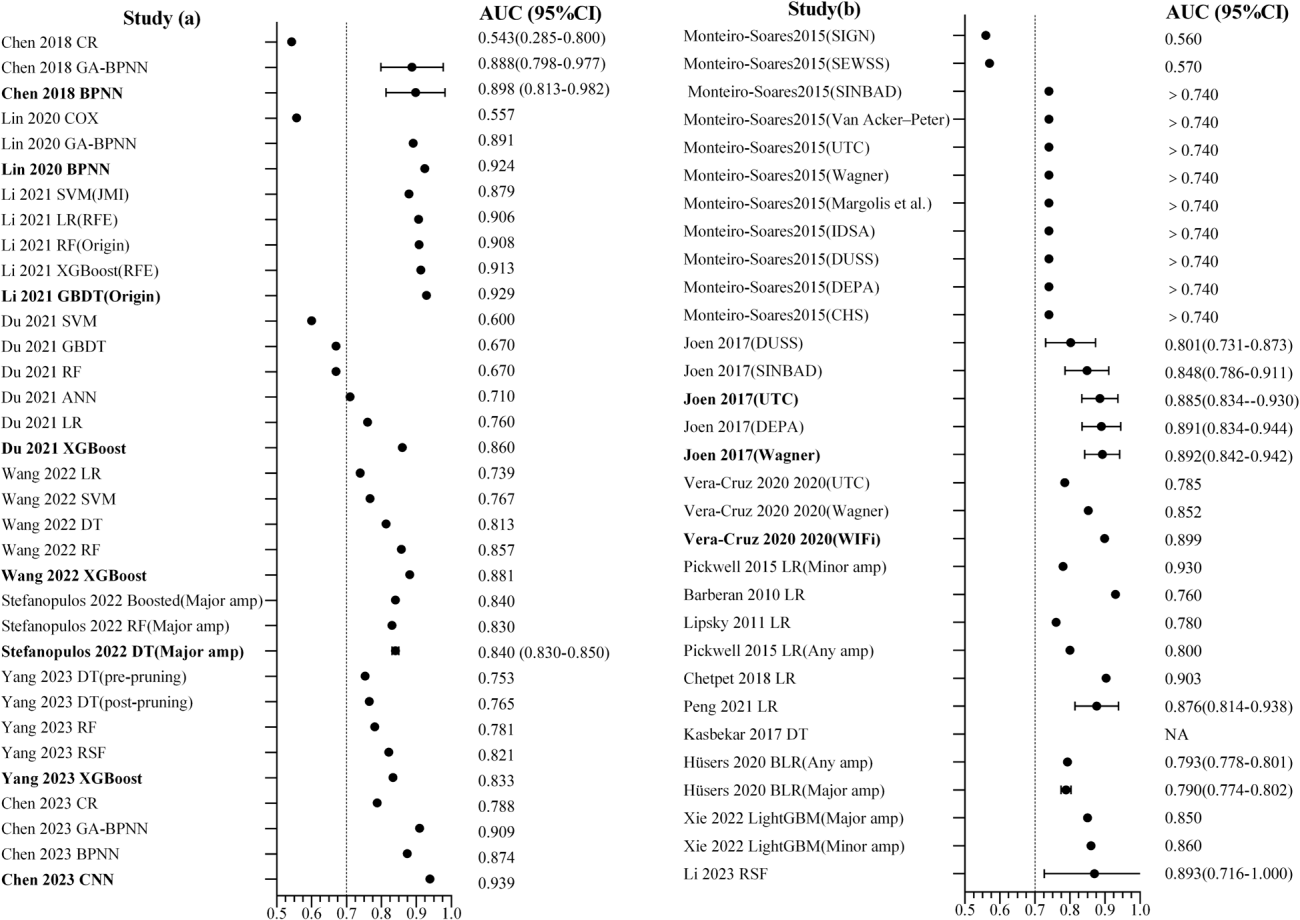
The values of area under the curve. Study **a** AUC values based on multiple machine methods; Study **b** AUC values of a single model development method and model validation study. AUC in the figure indicated model validation; without validation, assess modeling performance. Bold font referred to the preferred model by the study. We considered AUC = 0.5–0.7 as poor discrimination, 0.7–0.8 as moderate discrimination, 0.8–0.9 as good discrimination, and 0.9–1.0 as excellent discrimination. *AUC* area under the curve, *GA-BPNN* Genetic Algorithm-Based Backpropagation Neural Network, *SVM* support vector machine, *RFE* recursive feature elimination, *RF* Random Forest, *XGBoost* extreme gradient boosting, *GBDT* gradient boosting decision tree, *ANN* artificial neural network, *DT* decision tree, *Amp* amputation, *CNN* convolution neural network, *LightGBM* Light Gradient Boosting Machine, *BLR* Bayesian logistic regression, *scoring system* SIGN, Scottish Intercollegiate Guidelines Network, *SEWSS* Saint Elian Wound Score System, *SINBAD* site, ischemia, neuropathy, bacterial infection, and depth, *DUSS* diabetic ulcer severity score, *DEPA* depth of the ulcer, extent of bacterial colonization, phase of ulcer and association aetiology, *CHS* curative health services wound grade scale

**Fig. 3:**
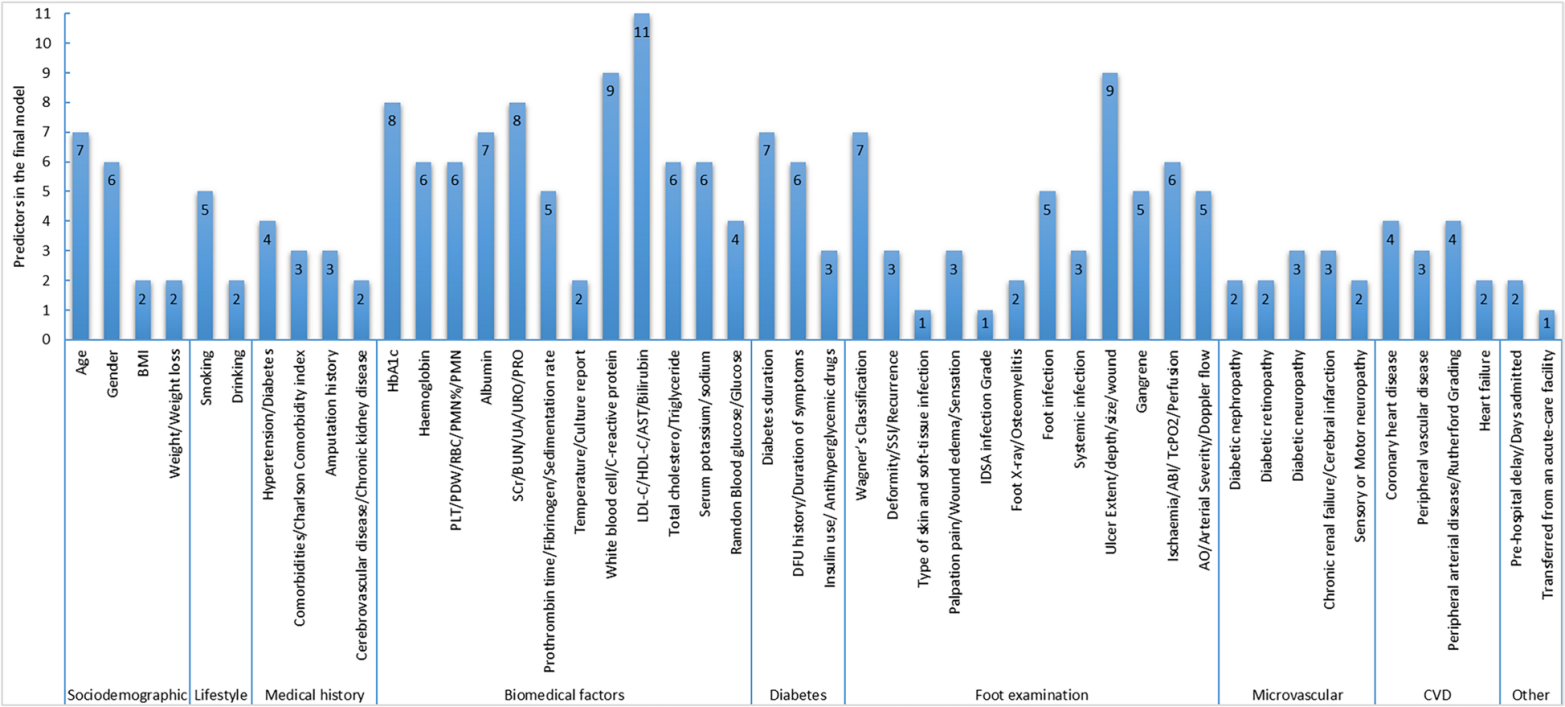
Map of final predictors distribution. *PLT* platelet, *PDW* platelet distribution width, *RBC* red blood cell, *PMN* neutrophil, *SCr* serum creatinine, *BUN* blood urea nitrogen, *UA* uric acid, *URO* urobilinogen, *PRO* urine protein, *LDL-C* low-density lipoprotein cholesterol, *HDL-C* high-density lipoprotein cholesterol, *AST* aspartate aminotransferase; *SSI* surgical site infection, *IDSA* infectious diseases society of America, *ABI* ankle-brachial index, *TcPO*_*2*_ percutaneous oxygen partial pressure, *AO* arterial occlusion

The AUC (areas under the curve) values for each model were shown in Fig. 2. The reported AUCs in the model development research ranged from 0.790 to 0.939. In the validation model studies, with the exception of SIGN (Scottish Intercollegiate Guidelines Network) and SWESS (Saint Elian Wound Score System), which demonstrated AUC values below 0.6 [18], the remaining models displayed AUC values exceeding 0.7, signifying robust model performance. Kasbekar et al. [37] exclusively reported model accuracy, while another study focused solely on model sensitivity and specificity [18]. Remarkably, only one study employed decision curve analysis (DCA) to evaluate the threshold probability of model benefit [31], while five studies opted for the Hosmer–Lemeshow (H–L) goodness-of-fit test or calibration curve to assess model calibration [23, 28, 31, 39, 41]. Across three model validation studies involving 11 scoring systems, Wagner’s Classification and UTC (University of Texas classification) emerged as the most frequently validated systems, featured in all three studies. The final models incorporated between 2 and 37 factors, comprising a total of 88 predictive factors across eight categories: sociodemographic, lifestyle, biomedical factors, diabetes-related factors, foot examination, microvascular complications, cardiovascular disease, and others. Noteworthy risk factors, recurring more than 5 times in multivariate models, included age, gender, HbA1c, hemoglobin (HGB), white blood cell count (WBC), low-density lipoprotein cholesterol (LDL-C), diabetes duration, and Wagner’s Classification. The distribution of predictors was illustrated in Fig. 3. For further details, please refer to Supplementary material (Table S1).

### Risk of bias and applicability evaluation

All studies were evaluated to have a high risk of bias, indicating methodological issues in either the development or validation of the model. In the participant domain, eight studies were identified as having a high risk of bias, primarily due to their reliance on retrospective data or the potential lack of full representation of the model’s target population within the selected subject [17, 24, 29–32, 39, 41]. Some studies exclusively enrolled patients with DFU, potentially leading to an overestimation of the model’s performance [17, 29, 32]. In terms of predictors, six studies were classified as having a high risk of bias, while two were deemed unclear. The primary factors contributing to bias were identified as follows: in studies utilizing data from multicenter healthcare institutions, subjective predictors such as peri-wound edema, ulcer size, and ulcer depth necessitate measurements from personnel with varying clinical experience or qualifications, thereby increasing the risk bias within the predictors [38]. Retrospective studies often lacked specification regarding the type of specification [29, 30, 41] or failed to ensure blinding of predictor evaluators [24, 31, 32], resulting in classifications of “unclear” or “high risk of bias.” Additionally, certain predictors, such as culture report and osteomyelitis, may exhibit a time lag in their results compared to other information [30, 37], which fails to meet the requirement of having “all predictors available at the time the model is intended to be used.” In the realm of outcomes, three studies included the history of amputation as predictors [23, 36, 39], leading to potential duplication in outcome indicators, thereby possibly inflating the model’s performance and warranting a rating of “high risk”. Furthermore, six studies had uncertain forecast times. Given that the criteria for DF amputation typically involve a comprehensive evaluation of multiple factors, including clinical symptoms, signs, imaging examinations, and blood circulation, which often require subjective judgment, the utilization of data from multicenter retrospective studies may lack unified criteria for outcome indicators. Therefore, the signal problem “Was the outcome defined and determined in a similar way for all participants?” for the four studies was ruled “PN” [28–30, 39].

In the analysis domain, all 20 studies were found to have a high risk of bias. Among them, 16 out of 17 model development studies had insufficient sample sizes, with only one study meeting the recommended guideline of having more than 20 EPV [30]. Additionally, one model validation study had a sample size of less than 100 [33]. Eight studies converted continuous variables into categorical ones, either entirely or partially, using arbitrary rules for categorization [29, 30, 32, 33, 36, 38, 39, 42]. This approach simplified the relationship between variables, potentially introducing subjective bias or information loss and reducing the model’s flexibility. Li [24] employed Z-score standardization to preprocess continuous variables, aiding in dimensionality reduction, mitigating the influence of outliers, and enhancing the model’s convergence speed. Regarding missing data handling, six studies reported the number of missing persons or values [17, 18, 24, 35, 36, 41], but only two of them detailed specific treatment methods. Specifically, Yang [41] removed cases with over 40% missing values and imputed missing quantitative data using the median. Conversely, Li’s strategy involved deleting features with more than 60% missing data and filling remaining gaps using the median, mean, mode, and K-Nearest Neighbor (KNN) algorithm [24]. Moreover, two studies proceeded with modeling without conducting variable screening, opting instead for the full set of predictors [23, 32]. Another study failed to avoid selecting variables solely based on univariate analysis, overlooking potential interactions between features and possibly omitting key factors [40]. Additionally, nine studies did not consider competitive risk and time analysis in their models, potentially disregarding data complexity [17, 24, 29–32, 39, 40]. In terms of model performance evaluation, there’s a notable absence of standardized calibration assessment, with most studies concentrating solely on discrimination during both model development and validation stages. Notably, 75% of the studies did not report model calibration results, while only two studies omitted reporting model AUC values [18, 37]. Furthermore, four studies failed to address concerns related to model overfitting, underfitting, and optimism when evaluating model performance [35, 36, 38, 40, 41]. Additionally, six studies relied solely on internal validation methods, utilizing a single randomly split sample of participant data [22, 30, 34, 37, 39, 42]. Lastly, six studies did not provide clarity on whether the predictors and their weights in the final model were consistent with the reported multivariate analysis results [28, 32, 34, 37, 38, 42].

**Fig. 4:**
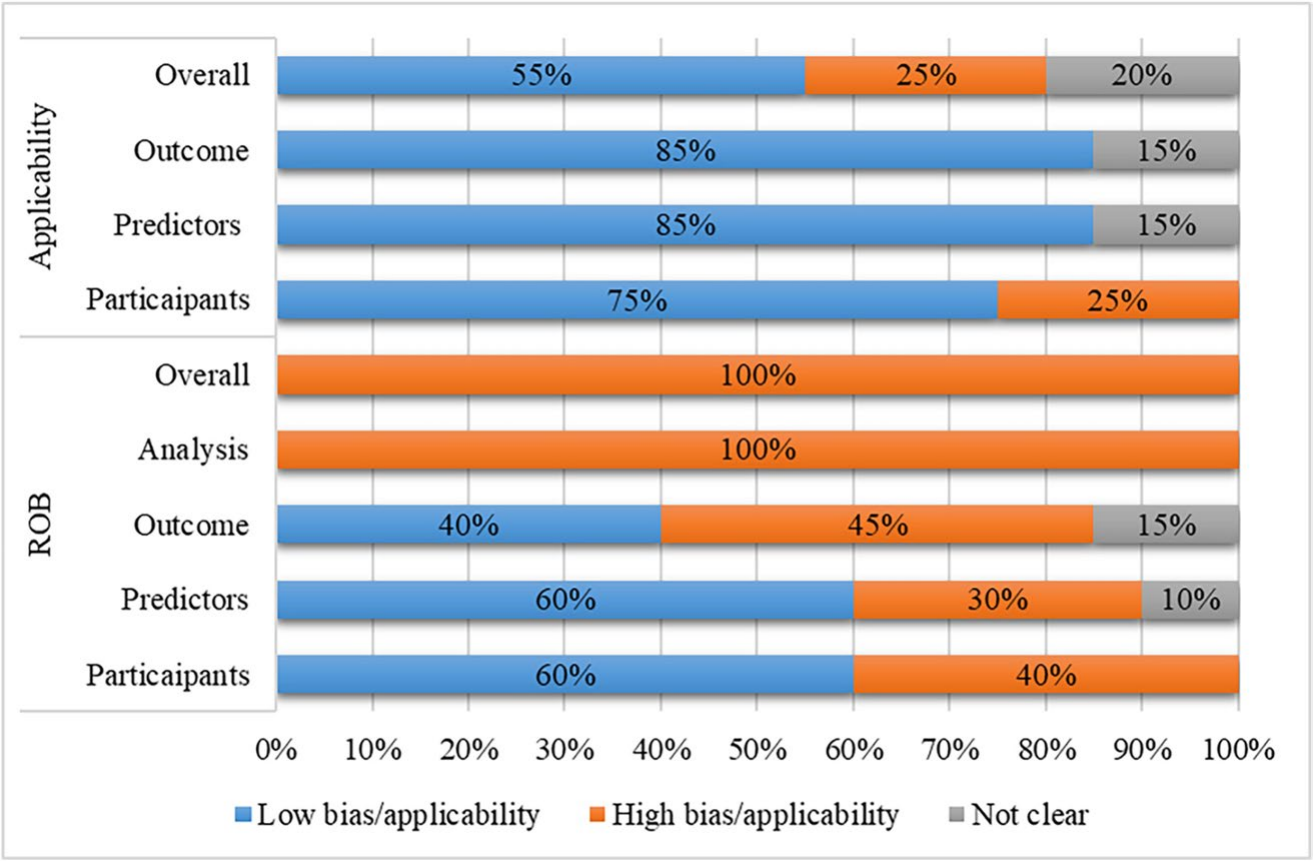
Percentage stacked chart regarding risk of bias and applicability assessment

Overall, five studies were deemed to have a high risk of applicability, while 11 were considered to have a low risk. In terms of participant domains in applicability, nine studies were categorized as high risk, primarily due to a lack of emphasis on DF across all degrees of ulceration groups. Concerning predictor domains, three studies were marked as unclear because the timing of prediction was not clearly reported, and there were uncertainties regarding the timing of predictor measurements. Regarding the outcome domain, five studies did not provide information on the predicted time of the outcome. Table 3 and Fig. 4 showed the included literature’s risk of bias and applicability according to PROBAST analysis.

**Table 3:** PROBAST results of included studies

Study Author (year)	Study type	ROB				Applicability			Overall	
		Participants	Predictors	Outcome	Analysis	Participants	Predictors	Outcome	ROB	Applicability
Chen 2023	D + V	+	+	+	−	+	+	+	−	+
Li 2023	D	+	+	−	−	+	+	+	−	+
Yang 2023	D + V	−	?	?	−	+	?	?	−	?
Stefanopoulos 2022	D + V	−	−	−	−	+	?	?	−	?
Wang 2022	D + V	−	?	−	−	−	?	?	−	−
Xie 2022	D + V	+	+	−	−	+	+	?	−	?
Du 2021	D + V	−	−	−	−	−	+	+	−	−
Li 2021	D + V	−	−	−	−	+	+	+	−	+
Peng 2021	D + V	−	−	−	−	+	+	+	−	+
Hüsers 2020	D	+	+	+	−	+	+	+	−	+
Lin 2020	D + V	+	+	+	−	+	+	+	−	+
Vera-Cruz 2020	V	+	+	+	−	+	+	+	−	+
Chetpet 2018	D	+	+	−	−	+	+	+	−	+
Chen 2018	D + V	+	+	+	−	+	+	+	−	+
Jeon 2017	V	−	+	?	−	−	+	+	−	-
Kasbekar 2017	D + V	+	−	?	−	+	+	?	−	?
Monteiro-Soares 2015	V	+	+	+	−	−	+	+	−	−
Pickwell 2015	D	+	+	+	−	+	+	+	−	+
Lipsky 2011	D + V	−	−	−	−	+	+	+	−	+
Barberan 2010	D	+	+	+	−	−	+	+	−	−

ROB: risk of bias; D: development only; D + V: development and validation in the same publication; V: external validation; +: indicated low ROB/low concern regarding applicability; −: indicated high ROB/high concern regarding applicability; ?: indicated unclear ROB/unclear concern regarding applicability

## Discussion

The reported amputation incidence varies widely, mainly due to differences in ulcer severity among the study populations. Studies focusing on DFU patients typically report a higher amputation incidence compared to those focusing on DF patients alone. This discrepancy underscores the complexity of the disease progression, as the development of DF complications is influenced by numerous factors [43]. Therefore, early diagnosis and intervention are imperative. Previous studies have shown that many major amputations can be prevented through timely risk interventions, including optimal glycemic control, multidisciplinary cooperation, timely revascularization, active DF care and education, regular foot assessments, and early referrals for ulcerative lesions [44, 45]. Predictive models play a crucial role in stratifying the risk of DF amputation, enabling medical staff to prioritize interventions for high-risk patients and optimize resource allocation. With an increasing number of predictive models available, it’s crucial to select high-quality ones based on evidence recommendations to provide reliable screening tools for clinical practice. This study included 17 model development studies, comprising three minor amputation models, four major amputation models, and 13 any-amputation models. These models demonstrated moderate to good predictive performance in internal validation, with AUC values ranging from 0.790 to 0.939. Furthermore, 11 DF classification systems were evaluated in three external validation studies, reporting AUC values ranging from 0.560 to 0.899. Nonetheless, all the studies were appraised to have a high risk of bias, and five studies were considered of high concern with regard to applicability in line with PROBAST. The current landscape indicates a scarcity of robust prediction models, emphasizing the imperative for further high-quality studies to drive progress in this field.

The predictive model quantifies the degree of correspondence between the estimated probability and the potential probability of an event, with a primary focus on early risk identification and intervention. In contrast, the classification system places emphasis on a thorough evaluation of existing foot ulcers and offers guidance for treatment decisions, albeit susceptible to subjective assessment bias [46]. However, these two methodologies can synergistically complement each other in the comprehensive management of DF, ultimately enhancing the efficacy of both prevention and treatment measures [23]. Within the realm of clinical risk management, the integration of the classification system with other objective variables has shown potential in enhancing the predictive performance related to patient amputation. This review underscores the adoption of a combined approach in which nine developed models integrated the foot ulcer classification system for joint prediction, showcasing its superior performance compared to individual classification systems [22, 23, 32, 35–37, 40, 41]. Wagner’s classification (0–5 grade), renowned for its simplicity and ease of application, has historically received significant attention in earlier research endeavors [19]. Nonetheless, contemporary guidelines from the IWGDF now advocate for the consideration of the WIFi system, particularly for DF patients accompanied by PAD [46]. This framework serves as a tool for stratifying both the likelihood of healing and the risk of amputation. The WIFi system offers a more holistic assessment by evaluating the extent of tissue loss, ischemia, and foot infection across a spectrum ranging from none to severe. This systematic approach facilitates clinicians in accurately discerning and communicating the severity of DFU. Remarkably, the study conducted by Vera-Cruz et al. [33] highlighted the superior predictive performance of the WIFi system compared to Wagner’s Classification. It is imperative to underscore that the implementation of these assessment frameworks necessitates the expertise and training of specific assessors to ensure consistency in evaluation outcomes. In the clinical setting, healthcare professionals are afforded the flexibility to select the most suitable classification system tailored to the unique circumstances of each patient. This personalized approach enhances their ability to effectively predict DF amputation outcomes.

In addition to the classification system for DFU, commonly observed biomedical factors include HGB, LDL-C, HbA1c, and WBC. Previous studies have shown that elevated levels of WBC, C-reactive protein (CRP), and erythrocyte sedimentation rate (ESR) were associated with an increased risk of amputation [9, 47]. These markers of infection are often elevated in cases of foot infection or gangrene, with osteomyelitis also being a robust predictor of amputation, suggesting their potential indirect role in predicting amputation occurrence [48]. From another perspective, increased levels of acute-phase inflammatory markers may indicate impaired immune response, worsening peripheral circulation, or inflammatory processes and infections triggered by high blood sugar levels, contributing to the development of PAD and ultimately amputation [1]. Elevated HbA1c levels typically indicate poor blood sugar control, leading to microvascular and neuropathic complications that affect foot circulation and nerve supply, resulting in sensory loss, autonomic nerve dysfunction in the feet, and accelerated formation and deterioration of foot ulcers, thereby increasing amputation risk [43]. Similarly, an increase in LDL-C concentration in the blood may lead to abnormal deposition on cardiovascular artery walls, forming atherosclerotic plaques, vessel blockage, and subsequent peripheral arterial obstructive disease, all contributing to increased amputation risk [23]. Among sociodemographic variables, gender, age and diabetes duration emerged as frequent predictors, consistent with findings from systematic reviews by Shin et al. [49]. Behavioral differences between genders are believed to explain the higher risk of amputation among males [50]. Additionally, estrogen’s protective effect on females, particularly concerning cardiovascular factors, and potential gender differences in immune response may contribute to this disparity [51]. With advancing age, individuals experience a gradual decline in physiological functions, including metabolic capacity, immune function, and tissue repair, increasing the risk of amputation. Prolonged exposure to high blood sugar levels in diabetic patients results in damage to multiple systems and organs, including the nervous, vascular, and immune systems, elevating the risk of foot complications and ultimately leading to the formation of foot ulcers, which may necessitate amputation [1]. Overall, the consistent inclusion of predictive factors in the model provides readily accessible tools for healthcare professionals to promptly assess amputation risk.

Although some models demonstrated excellent predictive capabilities across the 20 studies, our assessment using PROBAST revealed that all studies were flagged for a high risk of bias, largely stemming from inadequate reporting in the outcome and analysis domains. Firstly, the majority of studies relied on retrospective data sources. While a few employed a prospective design, a significant number failed to implement a blinded approach to outcome determination and predictor information. To ensure objectivity in evaluation, results should be assessed by an independent evaluator. Additionally, patients with a history of amputation should be excluded from the study population to prevent overestimation of the model’s predictive performance. These individuals face a heightened risk of re-amputation due to various factors, including compromised blood vessels, exacerbated vascular lesions, increased infection risk, altered pain perception, and restricted mobility [52]. Secondly, adopting a uniform definition and measurement of predictors is essential to ensure consistent assessment among subjects. Particularly in subjective assessments, different assessors may introduce bias. This also applies to defining outcome indicators; where subjective judgments are involved, such as imaging or pathological findings, uniform evaluator training is necessary to mitigate individual differences. Finally, limited sample size posed another common issue, with an EPV of at least 10 widely accepted to minimize overfitting [53]. According to PROBAST standards, an EPV higher than 20 indicates lower overfitting risk in the model [26]. However, predictive models developed using ML techniques may require a larger sample size (EPV > 200) to adjust for overfitting [26]. Given the relatively low incidence of amputation and numerous candidate predictors, only one study in this review met the EPV criteria of PROBAST [30], while two studies met the EPV criteria of 10–20 [35, 39]. Furthermore, within the existing evidence base, many model studies inadequately report or mishandle missing data, with failure to report being more common than simply excluding missing data. Future research should employ appropriate missing data processing methods, such as multiple imputation, and ensure their transparent reporting in studies [26].

In summary, the lack of calibration evaluation in most models highlights a common issue in predictive modeling research and contributes to the high risk of bias in these models. Therefore, the Transparent Reporting of a multivariable prediction model for Individual Prognosis or Diagnosis (TRIPOD) statement recommends employing calibration diagrams, calibration curves, or H–L tests to assess model calibration [54]. Despite most research models have good predictive performance built by different algorithms, having the best predictive performance indicator does not necessarily mean good clinical applicability [55]. In this study, only Peng et al. [31] assessed the actual application effect of the DCA assessment model under different risk thresholds. Consequently, the clinical utility and scalability of these models have been called into question. Moving forward, emphasis should be placed on the generalizability of the models. While considering model accuracy, selecting the appropriate model based on clinical applicability and practical convenience is crucial, while avoiding excessive pursuit of statistical optimization.

## Study limitation

There are certain potential limitations to this study. Firstly, our inclusion criteria encompassed studies published in English or Chinese, potentially constraining the applicability of the findings to populations that speak different languages. This limitation might necessitate adaptations when implementing these models in diverse regions with varying linguistic backgrounds. Secondly, the study focused on a population of patients with DF or DFU and did not include predictions of amputation due to only the diabetic stage or peripheral arterial disease. In addition, quantitative synthesis and analysis of the overall model performance were not conducted due to methodological differences of original literature and transparency of data reporting. Finally, the complexity of the internal structure of the included literature, mostly based on ML methods, presents a challenge in explaining the prediction basis of the model, limiting its interpretability in clinical practice to some extent. Future research can utilize visual tools or employ local interpretative approaches to demonstrate or explain key features, weights, and decision paths of the model, enhancing trust and understanding of the model’s predictions and making them more applicable to clinical practice. Moreover, ML algorithms represent a novel and rapidly emerging approach for predicting patient outcomes. Unfortunately, reproducing these predictive models was not feasible in this study. Nevertheless, these findings are promising and warrant further investigation in future research.

## Conclusion

DF amputation risk prediction models demonstrated good discrimination and reasonable applicability. However, they were hindered by significant methodological limitations, introducing high bias risks that may potentially undermined model performance and clinical utility. Future model development studies should adhere to PROBAST guidelines as much as possible to reduce bias risks, and Hosmer should be employed to regulate the reporting process of predictive models [56]. Machine learning-based models could benefit from upcoming TRIPOD-AI [57] guidelines to further standardize scientific rigor in this field. Additionally, future research should focus on assessing the clinical utility of prediction models, balancing the pros and cons of medical interventions, and conducting multicenter, large-sample external validation to evaluate model applicability.

### Supplementary Information

Supplementary Material 1.

## Data Availability

The data that support the findings of this study are available from the corresponding author upon reasonable request.

## Abbreviations

DM: Diabetes mellitus
DF: Diabetic foot
DFU: Diabetic foot ulcers
IWGDF: International Working Group on the Diabetic Foot
TUC: University of Texas classification
WIFi: Wound, Ischaemia, Foot infection
CNKI: China National Knowledge Infrastructure
CBM: Chinese Biomedical Literature Database
VIP: Weipu
MeSH: Medical subject headings
T2D: Type 2 diabetes
CHARMS: Critical Appraisal and Data Extraction for Systematic Reviews of Prediction Modelling Studies
PROBAST: Prediction Study Risk of Bias Assessment Tool
PAD: Peripheral artery disease
PRISMA: Preferred Reporting Items for Systematic Reviews and Meta-Analyses
EPV: Events per variable
ML: Machine learning
RF: Random Forest
XGBoost: Extreme gradient boosting
SVM: Support vector machine
SHAP: SHapley Additive Explanations
AUC: Areas under the curve
SIGN: Scottish Intercollegiate Guidelines Network
SWESS: Saint Elian Wound Score System
H–L: Hosmer–Lemeshow
UTC: University of Texas classification
HGB: Hemoglobin
WBC: White blood cell count
LDL-C: Low-density lipoprotein cholesterol
KNN: K-Nearest Neighbor
CRP: C-reactive protein
ESR: Erythrocyte sedimentation rate
TRIPOD: Individual Prognosis or Diagnosis
