# Supplementary material for: Prediction models for amputation after diabetic foot: systematic review and critical appraisal
Source: Diabetol Metab Syndr. 2024 Jun 10;16:126. doi: 10.1186/s13098-024-01360-6 (PMC11163763; doi:10.1186/s13098-024-01360-6)
Supplement: Supplementary file 1 — Supplementary Material 1. [file 13098_2024_1360_MOESM1_ESM.docx]

**Strategies in English databases**

| PubMed 2023.11.22 | | Results |
| --- | --- | --- |
| #1 | "diabetes mellitus"[MeSH Terms] OR "DM"[Title/Abstract] OR "diabetic*"[Title/Abstract] OR "diabetes"[Title/Abstract] OR "diabetic foot"[MeSH Terms] OR "foot ulcer"[Title/Abstract] OR "DFU"[Title/Abstract] | 887,686 |
| #2 | "amputation, surgical"[MeSH Terms] OR "amputat*"[Title/Abstract] | 58,264 |
| #3 | "Validat*"[Title/Abstract] OR "validit*"[Title/Abstract] OR "Predict*"[Title/Abstract] OR "Rule*"[Title/Abstract] | 2,972,152 |
| #4 | "Decision*"[Title/Abstract] AND ("Model*"[Title/Abstract] OR "Clinical"[Title/Abstract]) | 254,871 |
| #5 | "Prognostic"[Title/Abstract] AND ("History"[Title/Abstract] OR "Variable*" [Title/Abstract] OR "Criteria"[Title/Abstract] OR "Score"[Title/Abstract] OR "Scores*"[Title/Abstract] OR "Characteristic*"[Title/Abstract] OR "Finding*"[Title/Abstract] OR "Factor*"[Title/Abstract] OR "Model*"[Title/Abstract]) | 300,374 |
| #6 | "risk score*"[Title/Abstract] OR "risk assessment*"[Title/Abstract] OR "algorithm*"[Title/Abstract] | 502,087 |
| #7 | #3 OR #4 OR #5 OR #6 | 3,612,482 |
| #8 | #1 AND #2 AND #7 | 1,956 |
| Web of Science 2023.11.22 | |  |
| #1 | TOPIC: diabetes mellitus OR DM OR diabetic* OR diabetes OR diabetic foot OR DM OR foot ulcer OR DFU NOT | 1,736,466 |
| #2 | TOPIC: amputat* | 85,527 |
| #3 | TOPIC: Validat* OR validit* OR Predict* OR Rule* | 9,917,585 |
| #4 | TOPIC: Decision* AND (Model* OR Clinical) | 1,011,993 |
| #5 | TOPIC: Prognostic AND (History OR Variable* OR Criteria OR Score OR Scores* OR Characteristic* OR Finding* OR Factor* OR Model*) | 497,176 |
| #6 | TOPIC: risk score* OR risk assessment* OR algorithm* | 5,435,700 |
| #7 | #3 OR #4 OR #5 OR #6 | 14,809,882 |
| #8 | #1 AND #2 AND #7 | 4,556 |
| EBSCO CINAHL Plus 2023.11.17 | |  |
| S1 | MH "diabetes mellitus" OR AB "DM" OR AB "diabetic*" OR AB "diabetes" OR MH "diabetic foot" OR MH "foot ulcer" OR AB "DFU" | 221,598 |
| S2 | MH "amputation, surgical" OR AB "amputat*" | 11,573 |
| S3 | AB "Validat*" OR AB "validit*" OR AB "Predict*" OR AB "Rule*" | 581,010 |
| S4 | AB "Decision*" OR AB "Model*" OR AB "Clinical" | 1,482,072 |
| S5 | AB "Prognostic" OR AB "History" OR AB "Variable*" OR AB "Criteria" OR AB "Score" OR AB "Scores*" OR AB "Characteristic*" OR AB "Finding*" OR AB "Factor*" OR AB "Model*" | 2,232,927 |
| S6 | AB "risk score*" OR AB "risk assessment*" OR AB "algorithm*" | 90,560 |
| S7 | S3 OR S4 OR S5 OR S6 | 2,758,000 |
| S8 | S1 AND S2 AND S7 | 2,550 |
| EMBASE 2023.11.24 | |  |
| 1 | 'diabetes mellitus'/exp OR 'diabetes mellitus' OR 'diabetic foot'/exp OR 'diabetic foot' OR 'foot ulcer'/exp OR 'foot ulcer' | 1,343,781 |
| 2 | dm:ti,ab,kw OR diabetic*:ti,ab,kw OR diabetes:ti,ab,kw OR dfu:ti,ab,kw | 1,275,700 |
| 3 | #1 OR #2 | 1,560,526 |
| 4 | 'amputation'/exp OR 'amputation' | 82,465 |
| 5 | amputat*:ti,ab,kw | 66,814 |
| 6 | #4 OR #5 | 88,069 |
| 7 | Validat*:ti,ab,kw OR validit*:ti,ab,kw OR Predict*:ti,ab,kw OR Rule*:ti,ab,kw | 4,003,935 |
| 8 | Decision*:ti,ab,kw AND (Model*:ti,ab,kw OR Clinical:ti,ab,kw) | 368,072 |
| 9 | Prognostic:ti,ab,kw AND (History:ti,ab,kw OR Variable*:ti,ab,kw OR Criteria:ti,ab,kw OR Score:ti,ab,kw OR Scores*:ti,ab,kw OR Characteristic*:ti,ab,kw OR Finding*:ti,ab,kw OR Factor*:ti,ab,kw OR Model*:ti,ab,kw) | 464,137 |
| 10 | 'risk score*':ti,ab,kw OR 'risk assessment*':ti,ab,kw OR algorithm*:ti,ab,kw | 655,168 |
| 11 | #7 OR #8 OR #9 OR #10 | 4,876,752 |
| 12 | #3 AND #6 AND #11 | 3,628 |
| Cochrane Library 2023.11.21 | |  |
| #1 | MeSH descriptor: [Diabetes Mellitus] this term only | 17,361 |
| #2 | (DM OR diabetic* OR diabetes):ti,ab,kw | 125,093 |
| #3 | MeSH descriptor: [Diabetic Foot] this term only | 1,458 |
| #4 | MeSH descriptor: [Foot Ulcer] this term only | 598 |
| #5 | #1 OR #2 OR #3 OR #4 | 125,131 |
| #6 | MeSH descriptor: [amputation, surgical] this term only | 591 |
| #7 | (amputat*):ti,ab,kw | 3,888 |
| #8 | #6 OR #7 | 3,888 |
| #9 | (Validat* OR validit* OR Predict* OR Rule*):ti,ab,kw | 173,428 |
| #10 | (Model* OR Clinical):ti,ab,kw | 1,049,174 |
| #11 | (Decision*):ti,ab,kw | 40,632 |
| #12 | #10 AND #11 | 30,152 |
| #13 | (History OR Variable* OR Criteria OR Score OR Scores* OR Characteristic* OR Finding* OR Factor* OR Model*):ti,ab,kw | 1,064,004 |
| #14 | (Prognostic):ti,ab,kw | 22,421 |
| #15 | #13 AND #14 | 19,038 |
| #16 | (risk score* OR risk assessment* OR algorithm*):ti,ab,kw | 128,656 |
| #17 | #9 OR #12 OR #15 OR #16 | 299,831 |
| #18 | #5 AND #8 AND #17 | 413 |

**Strategies in Chinese databases**

| CNKI 2023.11.21 | | Results |
| --- | --- | --- |
| #1 | 主题（糖尿病 + DM + 糖尿病足 + 足溃疡 + DFU） |  |
| #2 | 主题（截肢） |  |
| #3 | 主题 （预测模型 + 风险预测 + 风险评分 + 危险因素 + 列线图 + 风险因素 + 风险评估） |  |
| #4 | #1 AND #2 AND #3 | 265 |
| WAN FANG 2023.11.21 | | |
| #1 | 主题 （糖尿病 OR DM OR 糖尿病足 OR 足溃疡 OR DFU）模糊匹配 |  |
| #2 | 主题 （截肢）模糊匹配 |  |
| #3 | 主题 （预测模型 OR 风险预测 OR 风险评分 OR 危险因素 OR 列线图 OR 风险因素 OR 风险评估）模糊匹配 |  |
| #4 | 文献类型：期刊论文+学位论文 |  |
| #5 | #1 AND #2 AND #3 AND #4 | 615 |
| Chinese Biomedical Literature Database 2023.11.21 | | |
| #1 | "糖尿病"[常用字段:智能] OR "DM"[常用字段:智能] OR "糖尿病足"[常用字段:智能] OR "足溃疡"[常用字段:智能] OR "DFU"[常用字段:智能] |  |
| #2 | "截肢"[常用字段:智能] |  |
| #3 | "预测模型"[常用字段:智能] OR "风险预测"[常用字段:智能] OR "风险评分"[常用字段:智能] OR "危险因素"[常用字段:智能] OR "列线图"[常用字段:智能] OR "风险因素"[常用字段:智能] OR "风险评估"[常用字段:智能] |  |
| #4 | #1 AND #2 AND #3 | 330 |
| VIP 2023.11.21 | | |
| #1 | 题名或关键词 （糖尿病 OR DM OR 糖尿病足 OR 足溃疡 OR DFU）模糊匹配 |  |
| #2 | 题名或关键词（截肢）模糊匹配 |  |
| #3 | 题名或关键词（预测模型 OR 风险预测 OR 风险评分 OR 危险因素 OR 列线图 OR 风险因素 OR 风险评估）模糊匹配 |  |
| #4 | #1 AND #2 AND #3 | 56 |

Table S2 Performance and predictors of risk prediction models for included prediction models

| First author  year | Performance^§^ | | | | | Predictors in final model^§^ | Model presentation |
| --- | --- | --- | --- | --- | --- | --- | --- |
|  | Development | | Validation | | Calibration  method |  |  |
| Chen  2023 | -- | | AUC:0.939, Sensitivity:90.7%  Specificity:91.1% | | None | Wagner’s Classification, ABI, LDL-C, TcPO_2_ | Model equation |
| Li  2023 | AUC:0.963,  Sensitivity:100%  Specificity:75.0% | | AUC:0.893  Sensitivity:85.7%  Specificity:98.0% | | H-L test | PRO, PLT, ALB, Fib, WBC, GLU, AST, DM duration, BUN, RBC, Foot infection, HTN, HGB, URO, Insulin use, CHD, DFU history, UA, Smoking, CRF, Age, Drinking, Gender | -- |
| Yang  2023 | AUC:0.821, Sensitivity:86.1%  Specificity: 64.2%, Accuracy: 78.2%, Precision: 87.4%  F1-score: 0.867 | | None | | H-L test | CD, Wagner’s Classification, Fib, PDW, HGB | Model equation |
| Stefanopoulos  2022 | AUC: --, Sensitivity:76.1%  Specificity: 79.3% | | AUC: 0.840, Sensitivity:76.2%  Specificity: 79.4% | | None | Gangrene, PVD, Weight loss, Systemic infection, Osteomyelitis | Web-based calculator |
| Wang  2022 | -- | | AUC:0.881, Sensitivity:76.7%,  Accuracy: 81.4%, Precision: 84.6%, F1-score: 0.867 | | None | Random BG, DM duration, CVD, PAD, Smoking, ALB, SCr, CRP, DFU history. | Web-based calculator |
| Xie  2022 | Development was Not report | Minor validation  AUC:0.860, Sensitivity: 64.3%,  Specificity: 94.5%, Precision: 60.0%,  NPV: 95.4% | | Major validation  AUC:0.850, Sensitivity: 33.3%  Specificity: 97.3%, Precision: 50.0%, NPV: 94.9% | H-L test,  Calibration curve | Age, Gender, BMI, DM duration, Smoking, Pre-hospital delay, HTN, CHD, HF, CI, DN, DNP, DR, PVD, AO, Gangrene, DFU history, Amputation history, HbA1c%, Random BG, WBC, PMN%, HGB, K^+^, Na^+^, SCr, ALB, TC, TG, LDL-C, HDL-C, AHG, Insulin use, Wagner’s and WIFi Classification (Wound, Ischaemia, Foot infection) | Feature significance using SHAP |
| Du  2021 | -- | | AUC: 0.860, Sensitivity: 67.0%, Specificity: 86.0%, Accuracy: 80.0%, Precision: 67.0%, NPV: 86.0% | | None | Age, Gender, DM duration, Pre-hospital delay, CCI, DFU history, HTN, CHD, CI, DN, DNP, DR, HF, Gangrene, Systemic infection, Smoking, Alcohol, HbA1c%, HGB, WBC, ALB, TC, HDL-C, LDL-C, TG, K^+^, Na^+^, CRP, WIFi classification (Wound, Ischaemia, Foot infection) | -- |
| Li  2021 | AUC: 0.916, Sensitivity: 66.7%, Accuracy: 89.8%, Precision: 77.4% | | -- | | None | Age, PMN%, HGB, K^+^, Na^+^, SCr, HbA1c, CRP, ALB, TC, TG, LDL-C, HDL-C, Wagner’s Classification, Gender, PMN, WBC, BMI, Ulcer extent, DM duration, Gangrene, DFU history, Foot infection, Systemic infection, Recurrence, Pre-hospital delay, AO, Smoking, CCI, DN | Amputation risk assessment and prediction software |
| Peng  2021 | AUC: 0.876, Sensitivity: 86.0%, Specificity: 81.0%, Accuracy: 83.0% | | -- | | H-L test, Calibration curve | DM duration, PAD, HbA1c%, WBC, Fib | Nomograms |
| Hüsers  2020 | *Major*: AUC: 0.790  *Any-amputation*: AUC: 0.793 | | None | | None | PEDIS: Perfusion, Ulcer Extent, Depth, Infection, Sensation. | Model equation |
| Lin  2020 | -- | | AUC:0.891, Sensitivity: 100%, Specificity: 79.0% | | None | HbA1c%, Ulcer Extent, LDL-C | -- |
| Vera-Cruz  2020 | NA | | AUC:0.899 | | None | WIFi classification | Classifications system |
| Chetpet  2018 | AUC:0.903, Sensitivity: 75.0%, Specificity: 86.0% | | None | | None | Age, Sensory neuropathy, Motor neuropathy, Deformity, IDSA infection Grade, DM duration, HbA1c%, ABI, Amputation history, Ulcer depth, Rutherford Grading | Scoring system |
| Chen  2018 | AUC:0.898, Sensitivity: 100%, Specificity: 79.6% | | -- | | None | HbA1c%, Gangrene, LDL-C | -- |
| Joen  2017 | NA | | AUC:0.892, Sensitivity: 75.0%, Specificity: 94.0%, Accuracy: 85.0%, Precision: 93.0%, NPV: 80.0% | | None | Wagner’s Classification, UTC | Classifications system |
| Kasbekar  2017 | *Simple*  Accuracy: 96.4%  *Complex*  Accuracy: 100% | | *Simple*  Accuracy: 94.0%  *Complex*  Accuracy: 96% | | None | *Simple:* Doppler flow, Wagner’s Classification  *Complex:* Days admitted, Wagner’s Classification,  Foot X-ray, Doppler flow, HbA1c%, SCr, Duration of symptoms, Comorbidities, Age, PT, Random BG, DM history, ALB, Culture report, HGB, Bilirubin, Weight | -- |
| Monteiro-Soares^*^  2015 | NA | | Wagner’ Classifications (3+4)  Sensitivity: 86.0%, Specificity: 65.0% | | None | DEPA, DUSS, Wagner, SIGN classifications | Classifications system |
| Pickwell 2015 | *Amputation:* AUC:0.800  *Minor*: AUC: 0.780 | | None | | None | Gender, Pain on palpation, Periwound edema, Ulcer size, Ulcer depth, PAD | -- |
| Lipsky 2011 | C-statistic: 0.76 | | -- | | H-L test | CKD, Gender, Temperature, Age, Type of skin and soft-tissue infection, Amputation history, ALB, PVD, WBC, SSI, Transferred from an acute-care facility | Scoring system |
| Barberan 2010 | AUC:0.930, sensitivity:96.2%, specificity: 78.8% | | None | | None | Severity on arterial Doppler, Wagner’s Classification, ESR | Scoring system |

Comments: “--” “--"Indicated not reported

Abbreviations: ABI, Ankle-brachial index; LDL-C, Low-density lipoprotein cholesterol; TcPO_2_, Percutaneous oxygen partial pressure; HL, Hosmer-Lemeshow goodness of fit test; PRO, Urine protein; PLT, Platelet; ALB, Albumin; Fib, Fibrinogen; WBC, White blood cell; GLU, Glucose; AST, Aspartate aminotransferase; BUN, Blood urea nitrogen; RBC, Red blood cell; HTN, Hypertension; HGB, Haemoglobin; URO, Urobilinogen; UA, Uric acid; CRF, Chronic renal failure; CD, Cerebrovascular disease; PDW, Platelet distribution width; PVD, Peripheral vascular disease; BG, Blood glucose; CVD, Cardiovascular disease; PAD, Peripheral arterial disease; SCr, Serum creatinine; CRP, C-reactive protein; BMI, Body mass index; CHD, Coronary heart disease; HF, Heart failure; CI, Cerebral infarction; DN, Diabetic neuropathy; DNP, Diabetic nephropathy; DR, Diabetic retinopathy; AO, Arterial occlusion; PMN, Neutrophil; TC, Total cholesterol; TG, Triglyceride; HDL-C, High-Density Lipoprotein Cholesterol; AHG, Antihyperglycemic drugs; NPV, Negative predictive value; WIFi: Wound, Ischemia, Foot infection; CCI, Charlson Comorbidity index; IDSA, Infectious diseases society of America; PT, Prothrombin time; CKD, Chronic kidney disease; SSI, Surgical site infection; ESR, Sedimentation rate.
